# Amyloid Deposits in the Ligamentum Flavum Related to Lumbar Spinal Canal Stenosis and Lumbar Disc Degeneration

**DOI:** 10.7759/cureus.26221

**Published:** 2022-06-22

**Authors:** Mustafa Al Yaseen, Haider Al Zahid, Sawsan Al-Haroon

**Affiliations:** 1 Trauma & Orthopaedics, West Hertfordshire Teaching Hospitals NHS (National Health Services) Trust, Watford, GBR; 2 Orthopaedics, Basra Teaching Hospital, Basra, IRQ; 3 Pathology, Basra Medical College, Basra, IRQ

**Keywords:** lumbar spine, amyloid, disc degeneration, stenosis, ligamentum flavum

## Abstract

Background: Amyloidosis is a protein conformational disorder, with distinctive features of accumulation of protein fibrils in different body tissues, causing a wide range of signs and symptoms. These amyloid fibrils are usually derived from about 30 different precursor proteins that have been identified. Although the most common tissue for their accumulation is cardiac, amyloidosis may appear in many other tissues, though rarely cause symptoms. One of these extracardiac tissues is the ligamentum flavum (LF).

Participants and Methods: Patients with lumbar spinal canal stenosis or lumbar disc degeneration, scheduled for surgery, were included in the study. A total of 17 LF specimens were obtained from 16 patients with lumbar spinal stenosis (two specimens were taken from two consecutive stenotic levels belonging to one patient), and 11 LF specimens were obtained from 11 patients with lumbar disc degeneration. Tissue biopsy was taken from the LF at the affected level and was stained with special immunohistochemical stain to detect transthyretin (TTR)-related amyloidosis (ATTR). The diameters of the lumbar canal and the LF thickness were measured at the affected level by a radiologist.

Results: This study includes 22 LF specimens. Male to female ratio was 5.4:4.6 with the mean age comparatively equal (M = 46 years for men and 48 years for women). The patients were divided into two groups: lumbar canal stenosis and lumbar disc degeneration. The result of the immunohistochemical stain towards TTR amyloid was positive in five out of 22 (22%) samples and all were from the stenosis group. The relationship of the LF thickness to the canal diameter in the positively stained stenosis group specimens was significant (*p *= 0.001). All the positive specimens were taken from levels L3−4 and L4−5.

Conclusion: There was a significant relationship between LF thickness and canal stenosis in the positively stained specimens (towards TTR amyloid) of the stenosis group. However, the disc degeneration group showed no relationship between canal diameter and LF thickness; moreover, all the specimens of that group stained negative. Middle-age patients with canal stenosis proved to have a significant relationship to amyloid deposit LF hypertrophy.

## Introduction

Lumbar spinal canal stenosis is a syndrome that greatly affects elderly people. Its signs and symptoms depend on the canal status and level and degree of compression [1]. The primary problem for patients is the pain that results from neurogenic claudication due to stenosis. In addition, it can impair walking and cause other forms of disabilities, especially in the elderly. Although symptomatic spinal canal stenosis has no accurate incidence or prevalence, it is considered the most common cause of spinal surgery in people older than 65 years [2]. There are many classifications for spinal canal stenosis; however, dividing them into congenital and acquired can provide clues about the nature of the canal compromise. Acquired canal stenosis is the most common type of canal compromise, which is usually attributed to the degenerative process of the spine as a consequence of aging [1].

The mechanism by which spinal canal stenosis affects nerves or causes neurological signs and symptoms is not well understood. Nevertheless, evidence suggests that the combination of stenosis, nerve compression, and lumbar extension reduces the cross-sectional area; hence, exerting compression on the venules around the nerve roots. Consequently, this leads to ischemic nerve impairment. This may explain the reversible nature of the symptoms when the patient bends forward [3-5].

Amyloidosis is denoted for very a diverse group of protein folding disorders. More than 30 different human proteins are shown to have a role in forming abnormal protein fibrils in vivo [6]. Amyloidosis mainly exists in two forms, local and systemic. The misfolded proteins can accumulate in most organs of the body but they are mainly isolated in the cardiac tissues, causing a life-threatening condition. They do accumulate in other organs throughout the body but their significance and effect are not well understood and have yet to be investigated [7].

Amyloid A (a member of the family apolipoproteins) is associated with high-density lipoproteins found in plasma. There are different isoforms of serum amyloid A found in the human body at different levels, depending on the inflammatory response. They are mainly formed in the liver [8]. Serum amyloid A (SSA) proteins have different functions, and they are usually secreted during the acute inflammatory phase. They function as a carrier for cholesterol to the liver and attract inflammatory cells towards the inflammatory site [9]. SSA1, SSA2, and SSA3 have been identified in humans. SSA4 and SSA5 have been recently identified in humans and mice, respectively [10].

Amyloid A detection in vitro occurs through a process of antigen-antibody reaction. Amyloid A antibody (mouse monoclonal antibody also known as immunohistochemical stain) reacts to antigens found in protein fibrils precipitated in various body tissues. This is a multi-step immunohistochemical process that involves binding the antibody-antigen, followed by a second antibody added to bind to the primary antibody, and finally the detection of bound antibodies by a colorimetric reaction [11].

## Materials and methods

In the presented study, all the specimens were taken from the affected level. All patients underwent lumbar spinal surgery in Basra Teaching Hospital, Basra, Iraq, and private hospital Ibn-Albaitar in Basra, Iraq. Tissue processing and paraffin blocking were done in the Basra Teaching Hospital Laboratory department, while the immunohistochemical staining was done in a private lab, Al-Bayan Lab in Basra, Iraq.

This study was approved by the Arab Board of Health Specializations Panel in Baghdad, Iraq (IRB No 11223). The patients were anonymized and the research was done on discarded, post-surgical tissues so no consent was needed from the patients.

All patients with lumbar disc degeneration and lumbar central spinal canal stenosis were included except patients with previous lumbar spine surgery, infection, trauma, anomalies, and tumour and metabolic bone disease.

Sections were stained using haematoxylin and eosin stain following the procedure of Avwioro (2011) [12]; followed by immunohistochemistry staining (Cuello, 1993) [13]. Slide preparation, as well as staining and detection of amyloid deposits under the microscope, were done by a pathologist. There was no prior Congo Red staining of the slides. All the slides were stained with a commercially available antibody solution (containing A rabbit polyclonal anti-transthyretin antibody) to detect transthyretin (TTR)-related amyloidosis (ATTR) within the ligamentum flavum (LF) tissue sample.

Slide preparation

Removal of Paraffin and Rehydration

The slides were placed in a 56-60 °C oven for 15 min ensuring that the oven temperature did not exceed 60 °C. The slides were then transferred to a xylene bath and two changes of xylene for five minutes each were performed. The excess liquid was shaken off and slides were rehydrated in two changes of fresh absolute ethanol for three minutes each. The excess liquid was shaken off and slides were placed in fresh 90% ethanol for three minutes. The excess liquid was again shaken off and slides were placed in fresh 80% ethanol for three minutes. The slides were then rinsed in running tap water gently for 30 seconds avoiding a direct jet, which may wash off or loosen the section. The slides were then placed in phosphate-buffered saline (PBS) wash bath for further rehydration (30 minutes at room temperature).

Antigen Retrieval: Unmasking of Antigen Microwave Retrieval

The slides were washed with deionized H2O and placed in a microwave-resistant plastic staining jar containing antigen retrieval solution. The microwave oven was operated for five minutes on high power (~700 watts) and it was ensured that the slides were still covered with retrieval solution or fresh solution was added and microwaving was repeated. This process was repeated two to three times. The slides were allowed to cool slowly at room temperature for at least 20 minutes.

Inactivation of Endogenous Peroxidase

The slides were placed on a flat level surface and were not allowed to touch each other. The sections were not allowed to dry out at any time. Enough drops of 3% hydrogen peroxide were added to cover the whole section. Incubation was done at room temperature for five minutes. Then slides were then placed in a PBS wash bath for two minutes.

Primary Antibody Reaction

The slides were allowed to drain and the excess fluid was shaken off with a brisk motion and each slide was carefully wiped around the sections. 100 μl primary antibody solution was applied to the appropriate slides, covering the tissue sections. Each slide was tilted in two different directions so that the liquid is spread evenly over the slide. Incubation was done for at least 60 minutes at 37°C in a humidified chamber. The slides were placed in a PBS wash bath for five minutes.

MRI measurements

**Figure 1:**
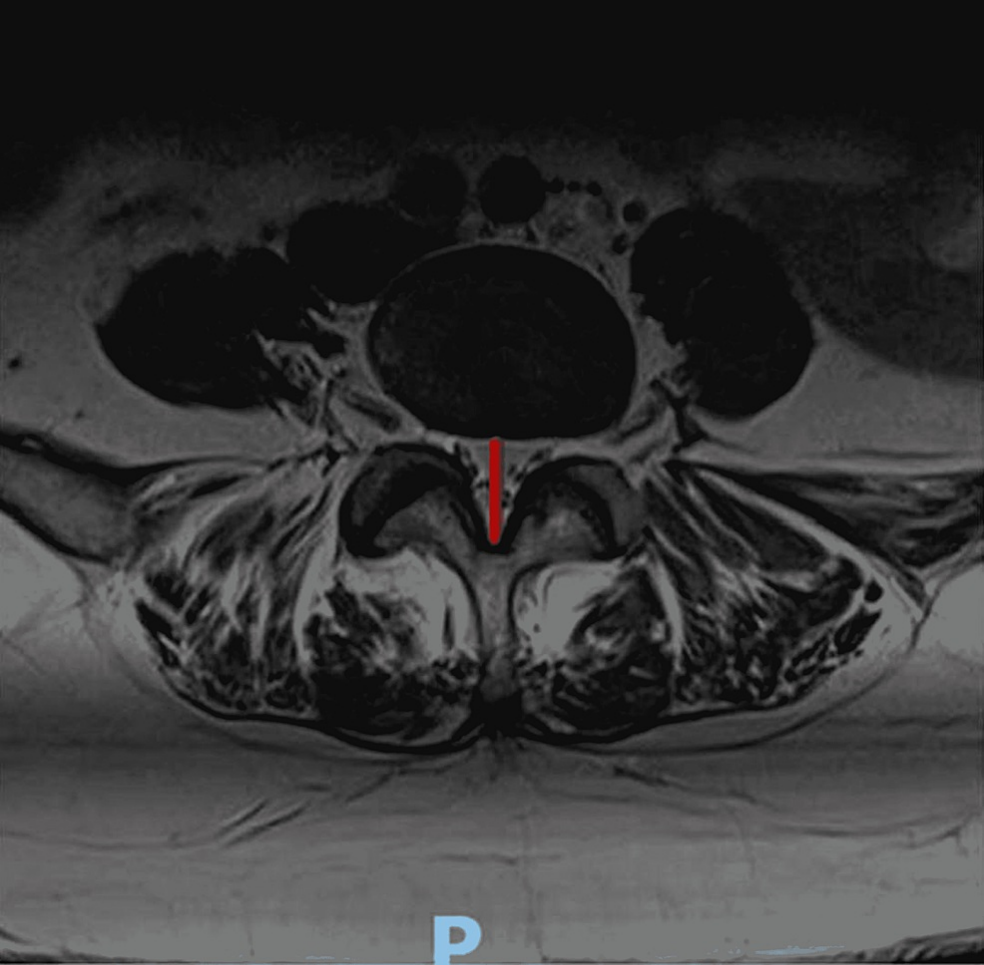
Lumbar canal measurement; red line shows the measurement of the canal P: posterior

The thickness of the LF and lumbar central canal diameter were measured at the T1-weighted axial section of the MRI, at the level of facet joints. MRI images were taken and measured three times by a radiologist on three different occasions. The average of the three measurements was taken as a final value (Figure 1). We received the information anonymized.

Statistics

Data were evaluated using Student's t-test and Fisher's exact test. All analyses were done using IBM SPSS Statistics for Windows, Version 24.0 (Released 2016; IBM Corp., Armonk, New York, United States). P-value < 0.05 was regarded as statistically significant.

## Results

In the presented study, 17 LF specimens were obtained from 16 patients with lumbar spinal stenosis (two specimens were taken from two consecutive stenotic levels belonging to one patient), and 11 LF specimens were obtained from 11 patients with lumbar disc degeneration. However, six specimens (all from the stenotic group) were corrupted during the processing. Only five LF specimens (of the 11 canal stenosis specimens), resected from 10 patients with spinal canal stenosis, were positive and reacted with the immunohistochemical stain (22.7% of the total number and 45.5% of the stenosis group specimens). All LF specimens resected from the 11 patients with lumbar disc degeneration were negative for the same stain (Table 1).

**Table 1:** Gender distribution of the results

Gender	Positive		Negative		Total
Male	1	8.3%	11	91.6%	12
Female	4	40%	6	60%	10
Total	5		17		22

Our study involved specimens from 12 males and 10 females, which represented 54.5% and 45.5% of the total number of specimens, respectively. One of the 12 males was found positive for amyloid stain (8.3 % of males), while four out of 10 females were found positive for the same stain (40% of females). The mean age of the patients was 46 years, with the age range of 25-70 years. The results were positive for amyloid in the sixth and seventh decade only, while negative in all other age groups (Table 2).

**Table 2:** Results according to age groups

Age group (in years)	Positive	Negative	Total
20-30	0	6	6
31-40	0	1	1
41-50	0	4	4
51-60	2	5	7
61-70	3	1	4
Total	5	17	22

In the present study, a significant relationship between the LF thickness and positive immunohistochemical was found. On the other hand, the relationship between central canal diameter (in stenosis patients only) and immunohistochemical staining results was not significant (p = 0.768). If we compare the relationship between the positive and negative specimens in the stenotic group only with the LF thickness, the result remains insignificant (p = 0.44; Table 3).

**Table 3:** Significance of relation between LF thickness and canal diameter with immunohistochemical stain * Measured only for stenosis patients All measures are Mean±SD LF: ligamentum flavum

Characteristic	Immunohistochemical positive	Immunohistochemical negative	P value
Mean LF thickness (mm) in both groups	4.64±0.16	3.21±0.81	0.001
Canal diameter (cm)*	1.3±0.19	1.42±0.26	0.768
LF thickness in the stenotic group (mm)	4.64±0.16	3.69±0.19	0.44

The positive cases were at levels L3−4 and L4−5 (three and two cases, respectively). Half of the specimens at level L3−4 were positive (three out of six), while only two out of 12 specimens were positive at level L4−5 (Table 4).

**Table 4:** Distribution of positively affected levels of lumbar spine

Affected level	Positive	Negative	Total
L2-3	0	1	1
L3-4	3	3	6
L4-5	2	10	12
L5-S1	0	3	3
Total	5	17	22

## Discussion

In the last few decades, there were many studies that reported the deposition of amyloid debris within different types of tissues throughout the body. However, it has been difficult to interpret the importance of such deposits as, in many instances, it has no significant clinical value. Amyloid deposits within a tissue are classified according to their precursor proteins; thus, the mere detection of amyloid without its original precursor protein is of small clinical value.

Zhong et al. reported that fibrosis is the main cause of LF hypertrophy, with subsequent spinal canal stenosis due to increased collagen contents and decreased elastin to collagen ratio [14-16]. However, subsequent studies proved the vital role of amyloid deposits (specifically the ATTR) in thickened LF and spinal instability [17].

On the other hand, Yayama et al. found that calcium pyrophosphate dihydrate crystal deposits (CPPD) play an important role in LF hypertrophy. They studied 270 LF specimens from patients with lumbar spinal stenosis and found that 35% of them tested positive for CPPD crystals [18].

With regard to the relationship between tissue amyloidosis and orthopaedic diseases, Yamamoto et al. had a good example [19]. Dialysis-related amyloidosis (DRA) is a common cause of systemic amyloidosis. The long-term, frequent haemodialysis affects patients adversely due to accumulated amyloid fibrils in the synovial membranes and osteoarticular sites, which cause carpal tunnel syndrome and spondyloarthropathy [19,20]. These findings, and others, motivated us to search for amyloidosis as a cause of spinal pathologies.

Spinal canal stenosis and lumbar disc degeneration are the most common spinal pathologies affecting different age groups, especially old age. Therefore, a search for its causes is urgent. Altun and Yüksel did a study on 71 patients with lumbar disc degeneration and lumbar spinal canal stenosis. They found that the LF histologic changes do not have any significant differences from their normal counterparts, unlike the stenotic group histologic changes, which may occur as a result of elastic fibre misalignment along with the development of calcification over time [21]. This finding supports our finding in that all the LF specimens taken from the lumbar disc degeneration group were negative, while five out of 11 (45.4%) specimens from the stenotic group were positive for immunohistochemical stain for amyloid.

Senile systemic amyloidosis is the most common type of amyloidosis affecting old age individuals [22]. However, the pathogenesis of senile systemic amyloidosis is not well understood and all the facts about this disease do not go beyond speculations and theories. Thereby, we cannot exclude that the accumulation of amyloid precursors within the LF may be part of senile systemic amyloidosis pathogenesis. Westermark et al. studied a specimen taken from the LF of 15 patients with lumbar spinal canal stenosis (seven males and eight females); he found that five (two females and three males) of the 15 specimens (33.3%) were positive for the immunohistochemical stain. The average age of the positive group was 82 years for females and 78 years for males [23]. We tested specimens taken from 11 patients (excluding the disc degeneration group) including nine females and two males. The results were positive for four females and one male (45.4%). The mean age of the positive group was 56.8 years for females and 52 years for males, despite being younger in comparison with the old age patients in Westermark et al.

Yanagisawa et al. studied 95 LF specimens obtained from 56 lumbar spinal stenosis patients and 19 lumbar disc degeneration patients. They used immunohistochemistry stain to detect TTR in these specimens. They found that all patients with lumbar disc degeneration tested negative for the stain, while 43 (45.2%) specimens tested positive from the stenosis group. They also found a significant relationship between LF thickness and the positively stained specimens (p <0.001). Moreover, they found a significant correlation between the lumbar spinal segmental instability and TTR-positive LF specimens taken from the affected level [17]. These results approximately approach our results regarding the correlation between amyloid positive specimens and LF thickness. Our study yielded a significant relationship between LF thickness and positively stained specimens (p <0.001), while all the specimens taken from lumbar disc degeneration group were negative. Regarding the result of the affected level, they found that two of three at level L3−4, four of 11 at level L4−5 level, and two of six at level L5−S1 were positive. This is unlike our results, which revealed three of six at level L3−4 and two of 12 at level L4−5 were positive for the immunohistochemical stain. There is a great similarity between the aforementioned study and our study in the finding of positive link between LF thickness and ATTR deposits; however, the level of spine segment affected is different, which can be attributed to the small number of the study group we examined.

Limitations

In our study, there were few limitations encountered at the start or throughout the duration of the study. Since it is self-funded, it is expensive to get more samples and stain with the immunohistochemical stain. Hence, we limited the number of the samples. Additionally, the process of getting the samples fixed on the slides (which was done in a national hospital at no charge) was not easy and led to the corruption of six samples.

## Conclusions

There is a relationship between the accumulation of amyloid precursors (ATTR) and LF hypertrophy in lumbar spinal canal stenosis in middle-aged patients. Nevertheless, this relationship is not evident when compared to young patients. The lumbar disc degeneration has no relationship to amyloid deposits within the LF.
